# miR-29a-3p orchestrates key signaling pathways for enhanced migration of human mesenchymal stem cells

**DOI:** 10.1186/s12964-024-01737-0

**Published:** 2024-07-17

**Authors:** Dayeon Kang, Taehwan Kim, Ga-Eun Choi, Arum Park, Jin Yoon, Jinho Yu, Nayoung Suh

**Affiliations:** 1 Department of Medical Sciences, General Graduate School, Soon Chun Hyang University, Asan, 31538 Republic of Korea; 2 Department of Pharmaceutical Engineering, College of Medical Sciences, Soon Chun Hyang University, Asan, 31538 Republic of Korea; 3 Asan Institute for Life Sciences, Asan Medical Center, Seoul, 05505 Republic of Korea; 4 Department of Pediatrics, Asan Medical Center, University of Ulsan College of Medicine, Seoul, 05505 Republic of Korea

**Keywords:** Cellular migration, miR-29a-3p, PTPRK, PTEN, Human mesenchymal stem cells

## Abstract

**Background:**

The homing of human mesenchymal stem cells (hMSCs) is crucial for their therapeutic efficacy and is characterized by the orchestrated regulation of multiple signaling modules. However, the principal upstream regulators that synchronize these signaling pathways and their mechanisms during cellular migration remain largely unexplored.

**Methods:**

miR-29a-3p was exogenously expressed in either wild-type or DiGeorge syndrome critical region 8 (DGCR8) knockdown hMSCs. Multiple pathway components were analyzed using Western blotting, immunohistochemistry, and real-time quantitative PCR. hMSC migration was assessed both in vitro and in vivo through wound healing, Transwell, contraction, and in vivo migration assays. Extensive bioinformatic analyses using gene set enrichment analysis and Ingenuity pathway analysis identified enriched pathways, upstream regulators, and downstream targets.

**Results:**

The global depletion of microRNAs (miRNAs) due to DGCR8 gene silencing, a critical component of miRNA biogenesis, significantly impaired hMSC migration. The bioinformatics analysis identified miR-29a-3p as a pivotal upstream regulator. Its overexpression in DGCR8-knockdown hMSCs markedly improved their migration capabilities. Our data demonstrate that miR-29a-3p enhances cell migration by directly inhibiting two key phosphatases: protein tyrosine phosphatase receptor type kappa (PTPRK) and phosphatase and tensin homolog (PTEN). The ectopic expression of miR-29a-3p stabilized the polarization of the Golgi apparatus and actin cytoskeleton during wound healing. It also altered actomyosin contractility and cellular traction forces by changing the distribution and phosphorylation of myosin light chain 2. Additionally, it regulated focal adhesions by modulating the levels of PTPRK and paxillin. In immunocompromised mice, the migration of hMSCs overexpressing miR-29a-3p toward a chemoattractant significantly increased.

**Conclusions:**

Our findings identify miR-29a-3p as a key upstream regulator that governs hMSC migration. Specifically, it was found to modulate principal signaling pathways, including polarization, actin cytoskeleton, contractility, and adhesion, both in vitro and in vivo, thereby reinforcing migration regulatory circuits.

**Supplementary Information:**

The online version contains supplementary material available at 10.1186/s12964-024-01737-0.

## Background

Human mesenchymal stem cells (hMSCs) are multipotent cells with remarkable therapeutic potential, largely due to their capacity to home to injury sites and facilitate tissue repair [1, 2]. Central to this homing ability is their migration, a complex and highly regulated process orchestrated by multiple signaling pathways. Despite significant advancements in understanding these pathways, many aspects of the molecular mechanisms governing hMSC migration remain unknown [3, 4].

Cell migration, including that of hMCSs, is fundamental to embryogenesis, angiogenesis, nerve growth, inflammation, wound healing, cancer metastasis, and many other physiological, developmental, and pathophysiological processes [5, 6]. It consists of a series of integrated steps: protrusion of the plasma membrane, attachment to the substrate, tension on actin filaments, and retraction of the cell tail. The dynamic nature of cell migration relies heavily on the rapid and reversible phosphorylation coordinated by kinases and phosphatases. For example, in the establishment and maintenance of cell polarity, phosphoinositide 3-kinases (PI3Ks) generate phosphatidylinositol (3,4,5)-trisphosphate (PtdIns (3,4,5)P_3_ or PIP_3_) at the front of the migrating cell, while phosphatase and tensin homolog (PTEN) removes them at the rear. Receptor-type protein tyrosine phosphatases (RPTPs) play significant roles in cell–cell and cell–matrix interactions by dephosphorylating multiple substrates [7]. They include protein tyrosine phosphatase receptor type F (PTPRF), which promotes cell adhesion to the matrix by regulating cyclin-dependent kinase 1 (CDK1) activity via c-Abl, while protein tyrosine phosphatase receptor type A (PTPRA) dephosphorylates the inhibitory Src Y527 site, leading to focal adhesion kinase (FAK) phosphorylation [8, 9]. Despite these insights, many of the upstream regulators of the multiple phosphatases necessary for coordinated cell migration have yet to be identified.

MicroRNAs (miRNAs) are small non-coding RNAs, typically around 22 nucleotides in length, that exert critical regulatory functions by targeting mRNAs for degradation or by modulating their translation by binding to the 3’-untranslated region (3’-UTR) [10]. Among the cellular processes regulated by miRNAs is cellular migration, which has been extensively studied in cancer [11]. For example, miR-21 promotes cancer cell migration and invasion by targeting tumor suppressor genes such as PTEN [12], while miR-34a suppresses cancer cell migration and invasion by targeting oncogenes including mesenchymal epithelial transition (MET) [13]. Some miRNAs, such as miR-20b and miR-182, exhibit dual roles in controlling cell migration, promoting or suppressing it depending on the context and specific signaling pathways involved [14, 15]. Similarly, a growing body of evidence indicates that specific miRNAs are crucial for the regulation of hMSC migration, but the precise molecular mechanisms remain to be fully elucidated.

In this study, we systematically investigated the effect of miRNA modulation on the migration of hMSCs by silencing DiGeorge syndrome critical region 8 (DGCR8), a crucial factor in miRNA biogenesis. The global loss of miRNAs resulted in significant migration defects in hMSCs. A bioinformatics analysis identified miR-29a-3p as an upstream regulator and demonstrated that its overexpression in DGCR8-knockdown hMSCs markedly rescued the migration defect. Our findings reveal that miR-29a-3p promotes cell migration by directly repressing two key phosphatases that act as negative regulators of cell migration: PTPRK, and PTEN. The ectopic expression of miR-29a-3p restored the polarization of the Golgi apparatus and actin cytoskeleton during wound healing. In addition, it modulated actomyosin contractility and cellular traction force by regulating the distribution and phosphorylation status of myosin light chain 2 (MLC2). miR-29a-3p also regulated focal adhesions (FAs) by modulating PTPRK and the FA marker paxillin. In immunocompromised mice, a substantial increase in the migration of hMSCs overexpressing miR-29a-3p towards stromal cell-derived factor 1 alpha (SDF-1α), a chemoattractant, was observed. These findings suggest that miR-29a-3p orchestrates the migration of hMSCs by regulating multiple downstream stages in vitro and in vivo.

## Methods

### Cell culture

This study was performed according to the guidelines of the Institutional Review Board of Soonchunhyang University (IRB no. 1040875-201708-BM-034). hMSCs were isolated from the adipose tissues of donors and cultured as described previously [16, 17]. They were used at passages 3 to 7. Human telomerase reverse transcriptase immortalized adipose-derived mesenchymal stem cells (hTERT-MSCs) (SCRC-4000; ATCC) were also used. hMSCs and hTERT-MSCs were cultured in Dulbecco’s modified Eagle’s medium (DMEM; Gibco) supplemented with 10% fetal bovine serum (Gibco), 100 units/mL penicillin, and 100 µg/mL streptomycin (Gibco) at 37 °C in a humidified atmosphere containing 5% CO_2_.

### Transfection

For transfection with small interfering RNAs (siRNAs), hMSCs were plated at 50–60% confluence in complete medium. The next day, the cells were transfected with ON-TARGETplus siRNAs including non-targeting control siRNA, human DGCR8 siRNA, human PTEN siRNA, or human PTPRK siRNA (Horizon Discovery) using Lipofectamine 2000 (Invitrogen) according to the manufacturer’s instructions. For miRNA transfection, 50 nM miR-29a-3p mimic (Horizon Discovery) was transfected using Dharmafect 1 (Horizon Discovery) 48 h after 50 nM siDGCR8 transfection according to the manufacturer’s instructions. For transfection of LNA miRNA inhibitors, cells were transfected with 50 nM miRCURY LNA miR-29a-3p inhibitor or miRCURY LNA miRNA inhibitor control B (Qiagen) using Dharmafect 1 (Horizon Discovery) for 24 h according to the manufacturer’s instructions.

### Transwell migration assay

A Transwell migration assay was performed using a 24-well cell culture plate with inserts that had an 8.0 μm pore size (Falcon). Transfected cells in serum-free medium were seeded to the upper chamber of the cell culture inserts, while complete medium was added to the lower chamber. For inhibitor treatments, transfected cells were seeded into the upper chamber in serum-free medium with 10 µM blebbistatin (blebb) (Sigma), 0.1 µM latrunculin A (Lat-A) (Sigma), 10 µM Y-27632 (Tocris), or 10 µM ML-7 (Sigma). When SDF-1α (PeproTech) was used as an attractant, transfected cells in complete medium were seeded into the upper chamber and complete medium with 100 ng/mL SDF-1α was added to the lower chamber. After 24 h of incubation, the cell culture inserts were fixed with 100% methanol, and stained with 0.3% crystal violet. Subsequently, cells that did not migrate were removed and the inserts were mounted on glass slides. Images were acquired using an Eclipse Ts2-FL inverted microscope (Nikon) and cells were enumerated using ImageJ software (NIH).

### Pathway analysis

**For Ingenuity pathway analysis (IPA):** Global gene expression profiling using the Illumina Human HT-12 v. 4.0 Expression BeadChip (GEO accession number: GSE149171) was conducted as previously described [18]. In that study, we identified differentially expressed genes (DEGs) as all genes showing differential expression (*P* < 0.05), among three sets of DGCR8-depleted and siGFP control hMSCs (2,383 transcripts). Then the DEGs were analyzed using IPA (Qiagen) to predict enriched molecular and cellular functions in DGCR8-depleted hMSCs and to identify upstream regulators. The miRNA target filter in the IPA was used for mRNA target prediction.

**For** **gene set enrichment analysis (GSEA)**: GSEA was performed using all mRNA profiling data (GSE149171). After performing 1000 permutations, gene sets with normalized enrichment scores ≥ 1 or ≤-1 and a false discovery rate (FDR) < 0.25 were considered enriched. An enrichment bubble plot was generated using SRplot (http://www.bioinformatics.com.cn/srplot). A GSEA enrichment map was generated using EnrichmentMap and the AutoAnnotate application in Cytoscape v. 3.10.1. For leading-edge analysis, the same DEGs served as input for GSEA, using the GO biological process gene sets. After the enrichment analysis, we selected migration-related downregulated gene sets in siDGCR8-transfected hMSCs for leading-edge analysis.

### Target prediction and luciferase reporter assay

The potential mRNA targets of miR-29a-3p were predicted using target prediction algorithms (TargetScan v. 8.0 and miRNA target filter in IPA software) and expression profiling data of GSE149171 [18]. To construct a luciferase reporter vector for the wild-type PTPRK 3’-UTR (1–1448), the 3’-UTR was amplified from genomic DNA from hMSCs by PCR. The PCR products were cloned into the psiCHECK-2 vector (Promega). A mutant PTPRK 3’-UTR was generated by site-directed mutagenesis using PrimeSTAR HS DNA Polymerase (TaKaRa) according to the manufacturer’s protocol. The sequences of the primers are listed in Table S1. Partial PTEN 3’-UTR fragments (504–855 or 1562–1913) with a miR-29a-3p binding site (or mutated sites) and *Xho*I/*Not*I recognition sites were synthesized. To construct a luciferase reporter, a synthesized fragment was first cloned into the pUCosmoAmp vector (Cosmogenetech). After digestion with *Xho*I and *Not*I, the fragment was inserted into the psiCHECK2 vector. The constructs were confirmed by sequencing.

HEK293T cells were seeded on 24-well plates (5 × 10^4^/well) to 60–70% confluence. The next day, they were transfected with 100 ng reporter constructs using Fugene 6 (Promega) and 50 nM miRNA mimics using Dharmafect 1 (Horizon Discovery). After 24 h of incubation at 37 °C, cells were lysed in Passive Lysis Buffer (Promega) and luciferase activities were measured using the Dual-Luciferase Reporter Assay System (Promega) on a VICTOR Nivo Multimode Microplate Reader (PerkinElmer).

### Quantitative real-time PCR

Total RNA was isolated from cultured cells using the Maxwell RSC miRNA Isolation Kit (Promega) on a Maxwell RSC Instrument (Promega) according to the manufacturer’s instructions. cDNA was prepared using random hexamers and SuperScript IV Reverse Transcriptase (Invitrogen). mRNA quantitative real-time PCR (qRT-PCR) was performed using Power SYBR green PCR Master Mix (Applied Biosystems) on a QuantStudio 6 Flex Real-Time PCR System (Applied Biosystems). The primers used for qRT-PCR are listed in Table S2. GAPDH was used as the loading control. To quantify the expression levels of miRNAs, total RNA was reverse-transcribed using the miScript II RT Kit (Qiagen) or miRCURY LNA RT Kit (Qiagen). qRT-PCR was performed using the miScript SYBR Green PCR Kit (Qiagen) or miRCURY LNA SYBR Green PCR Kits (Qiagen) according to the manufacturer’s instructions. SNORD68 or SNORD48 was used as the loading control. Each reaction was performed as at least three biological replicates with duplicate wells for each condition.

### Western blotting

Cells were lysed with RIPA buffer (Biosesang) containing Halt Protease and Phosphatase Inhibitor Cocktail (Thermo Scientific). Lysates were centrifuged at 12,000 rpm for 20 min at 4 °C and the protein concentrations in the supernatants were quantified using the Pierce BCA Protein Assay Kit (Thermo Scientific). Proteins were separated in 4–20% Mini-PROTEAN TGX™ Precast Protein Gels (Bio-Rad, Hercules, CA, USA) or by 10% SDS-PAGE. The proteins were transferred to nitrocellulose membranes (GE Healthcare) and blocked with 1% BSA or 5% BSA in TBST for 1 h at room temperature. After blocking, the membranes were incubated at 4 °C overnight with the appropriate primary antibodies: GAPDH mouse monoclonal antibody (sc-47724, Santa Cruz Biotechnology, Inc.), PTPRK mouse monoclonal antibody (sc-374315, Santa Cruz Biotechnology, Inc.), PTEN rabbit monoclonal antibody (9188, Cell Signaling Technology), Paxillin rabbit monoclonal antibody (ab32084, Abcam), and phospho-paxillin mouse monoclonal antibody (sc-365020, Santa Cruz Biotechnology, Inc.). GAPDH was used as the loading control. Next, the membranes were incubated for 2 h at RT with the following horseradish peroxidase (HRP)-conjugated anti-rabbit IgG (7074) and anti-mouse IgG (7076, both from Cell Signaling Technology) as secondary antibodies. The bands were visualized using the WesternBright ECL kit (Advansta). Images were acquired using the c300 chemiluminescent western blotting imaging system (Azure). The original blots are shown in Fig. S1.

### Wound healing assay

For wound healing assays, transfected hTERT-MSCs were seeded in either 24-well plates or µ-dishes (ibidi) and allowed to grow to confluence for 24 h. On the day of the experiment, the cells were scratched using a micropipette tip. For immunostaining, the cells were allowed to migrate for 4 h before fixation. Images were acquired using the Nikon Confocal AX (Nikon). Closure area, cell polarization, and actin orientation were evaluated using ImageJ software (NIH).

### Immunofluorescence

Cells were fixed in 4% paraformaldehyde (PFA) for 15 min and washed with PBS for 15 min. After washing, they were incubated in PBS supplemented with 0.1% Triton X-100 and 1% BSA for 1 h. Next, they were incubated at 4 °C overnight with the paxillin rabbit monoclonal (ab32084, Abcam) and phospho-myosin light chain 2 (pMLC2) (Thr18/Ser19) rabbit polyclonal (3674, Cell Signaling Technology) primary antibodies. They were washed with PBS for 15 min and incubated with goat anti-rabbit IgG Alexa Fluor 488 (Invitrogen) and/or rhodamine phalloidin (Invitrogen) secondary antibodies for 2 h in darkness. Finally, they were washed with PBS for 15 min, stained with 1 µg/mL DAPI (Sigma) for 15 min in darkness, and washed with PBS for 15 min. Images were acquired using the THUNDER Imager (Leica Microsystems Ltd.). Images were quantified using LAS X image-processing software (Leica Microsystems Ltd.) and ImageJ software (NIH).

To quantify cell polarization, migrating cells were fixed with 4% PFA, incubated in PBS with 0.1% Triton X-100 and 1% BSA for 1 h, and reacted with a GM130 rabbit antibody (12480, Cell Signaling Technology), rhodamine phalloidin (Invitrogen), and DAPI (Sigma). Images were acquired using the Nikon Confocal AX microscope (Nikon).

### Co-immunoprecipitation

hTERT-MSCs were lysed with Pierce™ IP Lysis Buffer (Thermo Scientific) with protease/phosphatase inhibitor cocktail (Cell Signaling Technology). Lysates were centrifuged at 12,000 rpm for 20 min at 4 °C and the protein concentrations in supernatants were quantified using the Pierce BCA protein assay kit (Thermo Scientific). The lysates were incubated overnight at 4 °C with magnetic beads and 1 µg paxillin antibody. Eluates were analyzed via western blotting using paxillin and PTPRK antibodies.

### Traction force microscopy

Traction force microscopy (TFM) was performed as described previously with minor modifications [19]. In brief, polyacrylamide (PAA) gels of 8.44 kPa stiffness were synthesized with 5% acrylamide (Sigma), 0.225% bis-acrylamide (Sigma), 1% ammonium persulfate (Sigma), 0.1% tetramethyl ethylenediamine (TEMED; Sigma), and 0.04% 0.5 μm diameter fluorescent polystyrene microspheres (Invitrogen) on glass-bottom dishes (SPL Life Sciences) [20]. Subsequently, PAA hydrogels were conjugated with 200 µg/mL rat tail collagen I (collagen I; Corning). Transfected hTERT-MSCs were seeded at 6 × 10^3^/dish and allowed to adhere for 48 h at 37 °C. Prior to imaging via the Eclipse Ts2-FL inverted microscope (Nikon), the plates were washed with DPBS. Images of the fluorescent microspheres and cells were acquired before and after cell detachment using 0.05% trypsin-EDTA (Gibco) for 15 min. Images were analyzed using a set of macros containing the Template Matching, Particle Image Velocimetry (PIV), and Fourier Transform Traction Cytometry (FTTC) plugins in ImageJ software (NIH) [19].

### Collagen contraction assay

After miRNA transfection, 3 × 10^5^ hTERT-MSCs were resuspended in 400 µL complete medium and embedded in 200 µL collagen I (Corning). After neutralization with 1 M NaOH, the mixture was seeded on a 24-well plate and incubated for 48 h in complete medium. The gels were detached from the wall and images were obtained 2 h after detachment. To determine the gel contraction value, the relative diameter of the well and the gel were measured using ImageJ software (NIH), and the percentage of contraction was calculated using the formula: 100 × (well diameter – gel diameter)/well diameter [21].

### In vivo cell migration assay

Six-week-old NOD/SCID male mice were purchased from Orient Bio Inc. The animal care and treatment procedures were approved by the Institutional Animal Care and Use Committee of Asan Medical Center (2021–12–265). hMSC migration was evaluated in vivo using a modified Matrigel plug assay [22, 23]. NOD/SCID mice were randomly divided into two groups: control miR-NC transfected hTERT-MSCs and miR-29a-3p-transfected hTERT-MSCs (*n* = 3 per group). Matrigel (Corning) with or without SDF-1α (100 ng/mL) was subcutaneously injected into the right and left dorsal side (one or two implants). After 2 h, transfected hTERT-MSCs (10^6^ in 50 µL PBS) were labeled with Qtracker™ 800 (Invitrogen) following the manufacturer’s protocol and injected subcutaneously at the center, equidistant from the implant plugs. Labelled cells were assessed at 0, 8, 24, and 46 h using the IVIS Spectrum In Vivo Imaging System (PerkinElmer) and ImageJ software (NIH).

### Statistical analysis

*p*-values were calculated using Student’s two-tailed t-test. GraphPad Prism 10.1.2 was used for data visualization and in the statistical analysis.

## Results

### miR-29a-3p rescues the migration defect in hMSCs following DGCR8-induced global miRNA depletion

**Fig. 1:**
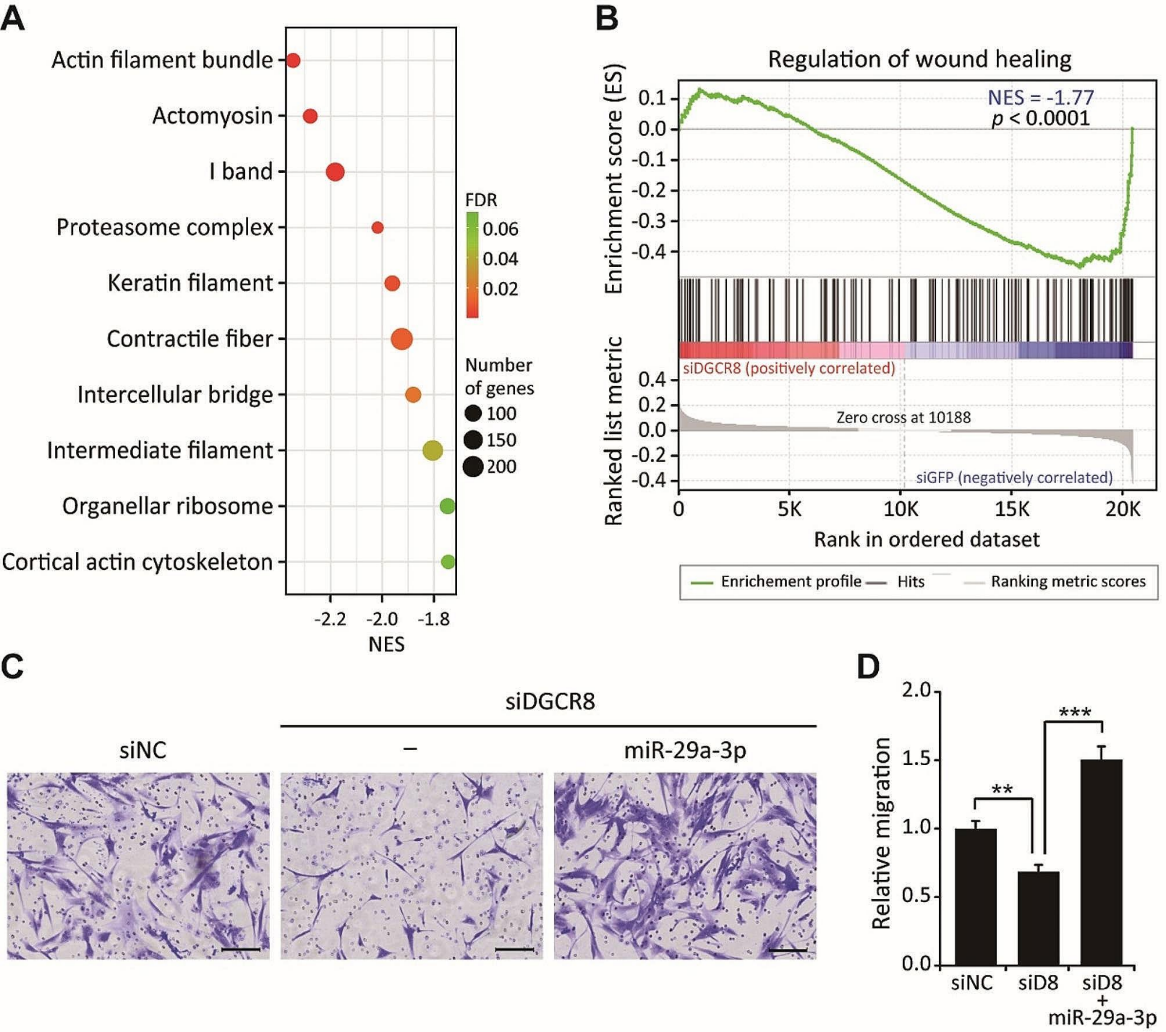
Overexpression of miR-29a-3p rescues the migration impairment caused by DGCR8 knockdown in hMSCs. (**A**) Bubble plot of the top 10 Gene Ontology (GO)_cellular components (CCs) significantly enriched in siDGCR8 vs. siGFP-transfected hMSCs by gene set enrichment analysis (GSEA). Each dot represents a GO_CC term; dot size and color indicate the number and false discovery rate (FDR), respectively. (**B**) A gene set of regulation of wound healing in siDGCR8 vs. siGFP-transfected hMSCs. Normalized enrichment scores (NES) and *p*-values are indicated. (**C**) Representative images of a Transwell migration assay. Scale bars, 100 μm. (**D**) Migrated cells were enumerated and statistically analyzed (error bars indicate the standard errors of mean of four experiments, **P < 0.01, ***P < 0.001 by Student’s two-tailed t-test). “siD8” indicates siRNA targeting DGCR8.

In a previous study we showed that knockdown of DGCR8, an indispensable component of miRNA biogenesis, results in abnormal cytokine secretion and the premature onset of cellular senescence in hMSCs [18, 24]. Considering the impact of miRNAs in hMSCs, we hypothesized that additional dysregulated functions are linked to global miRNA knockdown. This was explored by performing a GSEA for functional enrichment using our previously generated microarray profiling dataset with siGFP and siDGCR8-transfected hMSCs (GSE149171). Interestingly, the top 10 Gene Ontology (GO)_cellular components (GO_CCs) associated with DGCR8 knockdown were related to the actin filament bundle, actomyosin, contractile fiber, and cortical actin cytoskeleton (Fig. 1A). Specifically, a GO_biological process (GO_BP) related to the regulation of wound healing and including 127 genes was downregulated upon DGCR8 knockdown (Fig. 1B). These enrichment analyses suggested that DGCR8 knockdown correlates with cellular migration defects. This relationship was further examined in a Transwell migration assay, after confirming a significant reduction (0.47-fold) in the level of DGCR8 mRNA in siDGCR8-transfected hMSCs by quantitative real-time PCR (qRT-PCR) (Fig. S2A). Consistent with the enrichment results using GSEA, the migration of siDGCR8-transfected hMSCs was markedly reduced compared to siNC-transfected cells (0.69-fold, *P* < 0.01) (Fig. 1C).

Next, we set out to identify the miRNAs that could ameliorate the migration defect in siDGCR8-transfected cells. Candidate miRNAs were identified after investigating the upstream regulators linked to DEGs (siGFP vs. siDGCR8, *P* < 0.05, 2,383 transcripts from GSE149171) in DGCR8-knockdown versus control hMSCs using IPA software. The top five diseases and cellular functions associated with DGCR8 knockdown included cell death and disease, gene expression, cellular assembly and organization, cellular function and maintenance, and the cell cycle (Table S3). Because cellular migration is closely related to cellular assembly and organization and to cellular function and maintenance, the upstream regulators of those functions were considered. In total, 30 miRNAs were identified based on statistical parameters (*P* < 0.0001). Among these candidates, miR-29a-3p was of particular interest, due to conflicting reports concerning its role in migration and invasion in cancer cells [25–27] and because its effects on hMSC migration are largely unknown. The ability of miR-29a-3p to ameliorate the migration defect in siDGCR8-transfected cells was therefore examined by overexpressing miR-29a-3p in DGCR8-knockdown hMSCs. Transfection of a miR-29a-3p mimic led to a 31-fold increase in the miR-29a-3p level in siDGCR8-transfected cells according to qRT-PCR (Fig. S2B). Notably, the overexpression of miR-29a-3p significantly enhanced migration by 2.2-fold compared to siDGCR8-transfected hMSCs (Fig. 1C, D, *P* < 0.001). Compared to siNC-transfected cells, which have normal migration, the overexpression of miR-29a-3p resulted in a 1.5-fold improvement in migration (Fig. 1D, *P* < 0.01). To confirm the specific effect of miR-29a-3p on the impaired migration of siDGCR8-transfected hMSCs, we introduced an LNA miRNA inhibitor to wild-type hMSCs to suppress miR-29a-3p. The miR-29a-3p level decreased 0.09-fold according to qRT-PCR, resulting in a 25% decrease in the migration of LNA-miR-29a-3p–transfected hMSCs (Fig. S3). Taken together, these results indicated that the global loss of miRNAs in hMSCs leads to defects in cellular migration and miR-29a-3p can rescue the impaired migration of DGCR8-knockdown hMSCs, thus identifying miR-29a-3p as a key regulator of hMSC migration.

### Potential mRNA targets of miR-29a-3p in hMSCs exhibit enrichment in processes related to cellular assembly and organization

**Fig. 2:**
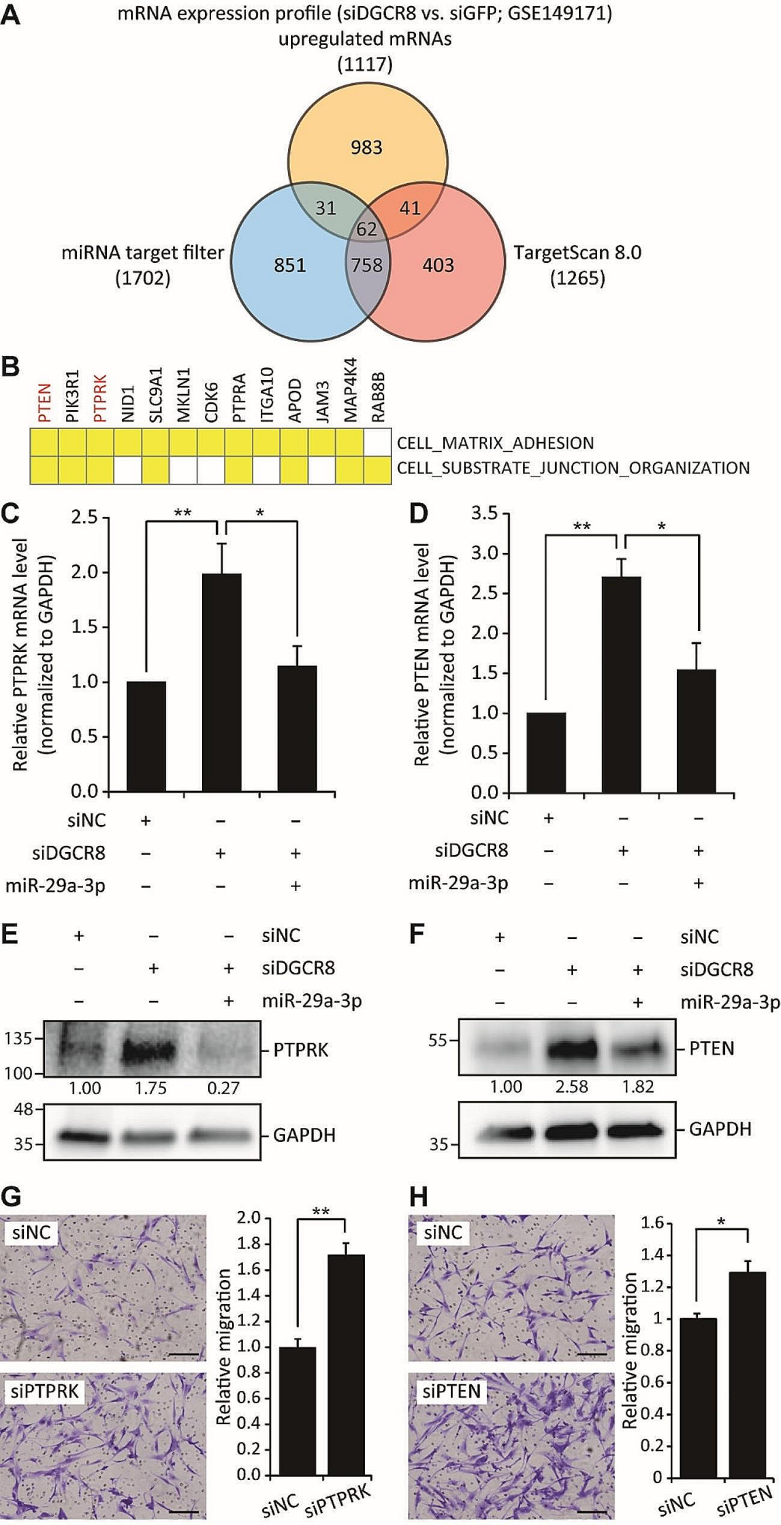
Putative mRNA targets of miR-29a-3p in hMSCs are enriched in processes associated with cellular assembly and organization. (**A**) Venn diagrams showing the numbers of putative targets according to miRNA target filter and TargetScan v. 8.0 and upregulated genes in DGCR8-knockdown cells. Sixty-two genes were identified by both target prediction algorithms and expression profiling (overlap in the Venn diagram). (**B**) Overlapping leading-edge genes in DGCR8-knockdown hMSCs. (**C**,** D**) mRNA levels of PTPRK (C) and PTEN (D) as determined by quantitative real-time PCR in DGCR8-knockdown hMSCs with or without miR-29a-3p overexpression. Data were normalized to the GAPDH mRNA level. (Error bars indicate standard errors of the mean of three and six experiments, *P < 0.05, **P < 0.01 by Student’s two-tailed t-test). (**E**,** F**) Immunoblot analysis of PTPRK (E) or PTEN (F) in DGCR8-knockdown cells with or without miR-29a-3p forced expression. GAPDH was used as the loading control. (**G**,** H**) Representative images of a Transwell migration assay in siPTPRK (G) and siPTEN (H) hMSCs. Scale bars, 100 μm. Migrated cells were quantified and statically analyzed (error bars indicate standard errors of the mean of four experiments, *P < 0.05, **P < 0.01 by Student’s two-tailed t-test)

The molecular mechanism by which miR-29a-3p enhances hMSC migration was investigated by performing target prediction analyses followed by experimental validation. Two miRNA target prediction tools and our previously published microarray gene profiling data of control and DGCR8-depleted hMSCs (GSE149171) were employed [18]. First, we compiled a list of putative miR-29a-3p targets using TargetScan v. 8.0 and the miRNA target filter in IPA software. This yielded 1,702 (miRNA target filter) and 1,265 (TargetScan v. 8.0) potential targets with a total of 2,146 non-redundant genes (Fig. 2A). In the context of mRNA expression profiles, we hypothesized that the levels of potential direct target mRNAs of miR-29a-3p would be upregulated in DGCR8-knockdown hMSCs compared to siGFP-transfected cells. Of those assessed, 1,117 genes were upregulated in siDGCR8-transfected hMSCs. We therefore focused on the 62 genes overlapping across all platforms to identify putative mRNA targets (Table S4). Pathway analysis using IPA revealed the top five molecular and cellular functions associated with these 62 genes (Table S5). Consistent with the impaired migration in DGCR8-knockdown cells, we found enrichment of functions related to cellular assembly and organization, including the orientation of the Golgi apparatus, formation of FAs, and formation of actin. Interestingly, the leading-edge subsets, which represent the core group of genes contributing the most to the enrichment signal in the GSEA, exhibited significant enrichment in several phosphatases (Fig. 2B). Because PTPRK and PTEN were predicted targets of miR-29a-3p and both phosphatases are negative regulators of cell migration, they were chosen for further investigation after confirming their upregulation in DGCR8-knockdown hMSCs at the mRNA (Fig. 2C, D) and protein (Fig. 2E, F) levels. To investigate whether miR-29a-3p influences the expression levels of PTPRK and PTEN in hMSCs, we overexpressed miR-29a-3p in a DGCR8-knockdown background. The PTPRK and PTEN mRNA (Fig. 2C, D) and protein levels (Fig. 2E, F) were downregulated upon miR-29a-3p overexpression. Next, we explored the effect of silencing PTEN and PTPRK on hMSC migration. Using siRNAs, we transiently silenced PTPRK and PTEN expression, which was confirmed by qRT-PCR and Western blotting (Fig. S4). Interestingly, knocking down PTPRK or PTEN enhanced the migration of hMSCs 1.7- and 1.3-fold, respectively (Fig. 2G, H), mimicking the effects of miR-29a-3p overexpression. In summary, we identified a subset of genes, including PTPRK and PTEN, as potential targets of miR-29a-3p; these genes were downregulated upon miR-29a-3p overexpression. Moreover, their knockdown enhanced cell migration.

### miR-29a-3p enhances hMSC migration by directly suppressing PTPRK and PTEN

**Fig. 3:**
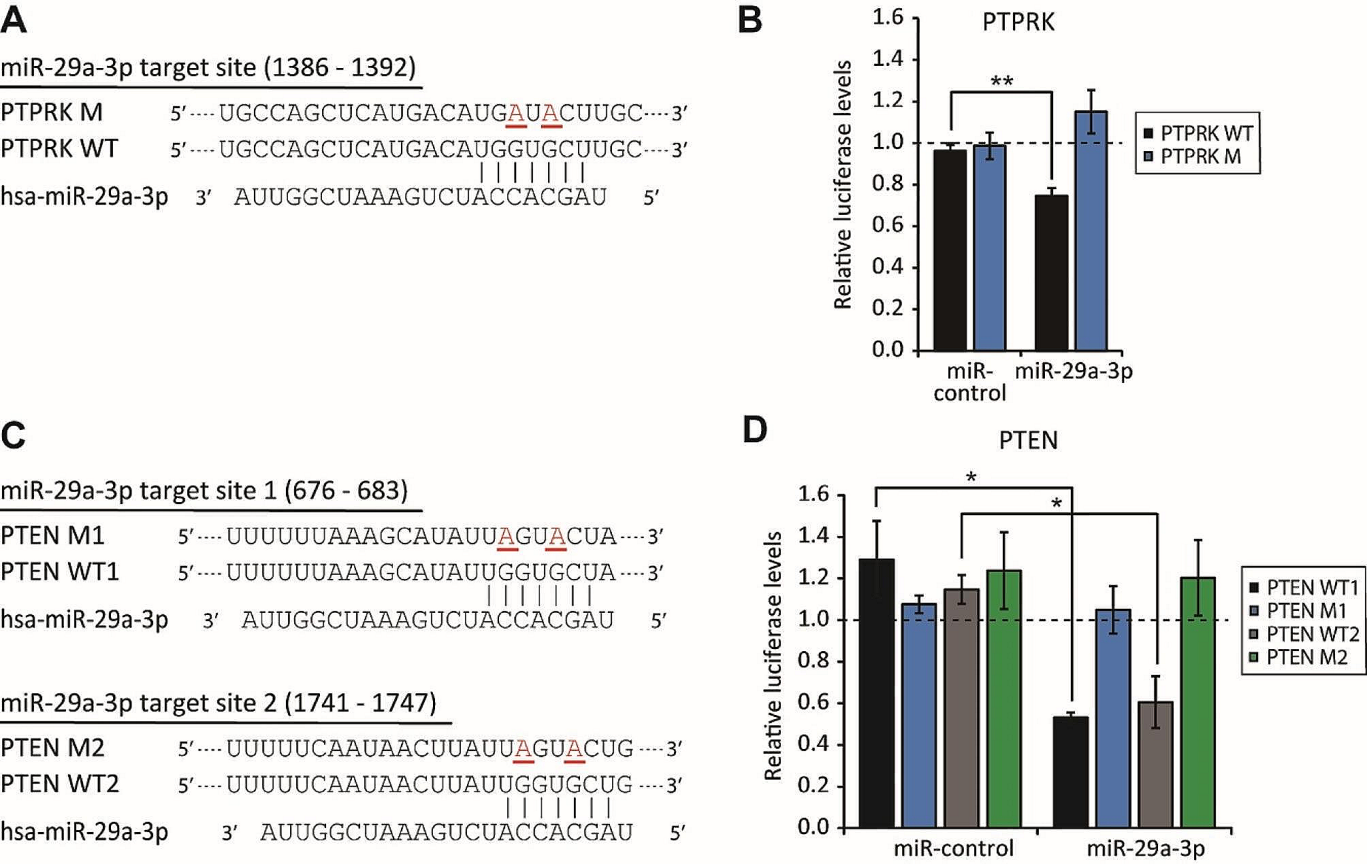
miR-29a-3p directly represses PTPRK and PTEN. (**A**,** C**) Schematic of miR-29a-3p binding sites in the 3’-UTR of PTPRK (A) and PTEN (C). Mutated nucleotides are underlined and highlighted in red. (**B**,** D**) Luciferase reporter assay. 293T cells were co-transfected with luciferase reporters carrying the wild-type or mutated 3′-UTR, as well as 50 nM negative control or miR-29a-3p mimics. Data were normalized to firefly luciferase expression. Values for mock-transfections set to 1 as denoted by the dashed line (error bars indicate standard errors of the mean of three experiments, *P < 0.05 and **P < 0.01 by Student’s two-tailed t-test)

To ascertain whether PTPRK and PTEN are direct targets of miR-29a-3p, we cloned the full 3’-UTR of PTPRK (1–1448) and the partial 3’-UTR of PTEN (504–855 or 1564–1913) downstream of the *Renilla* luciferase reporter gene. Subsequently, we cotransfected the reporter constructs with miR-29a-3p mimics into 293T cells (Fig. 3A, C). Compared to mock- and miR-control–transfected cells, luciferase activity significantly decreased 0.76-fold for PTPRK (Fig. 3B). For PTEN, the site 1 binding site (PTEN WT1) and site 2 binding site (PTEN WT2) were reduced 0.41- and 0.53-fold, respectively (Fig. 3D). Mutagenesis of the putative miRNA binding sites led to increased luciferase activity (Fig. 3B and D). In summary, our findings indicate that miR-29a-3p enhances hMSC migration in part by directly repressing PTPRK and/or PTEN.

### miR-29a-3p regulates cell polarization and migration during wound healing

**Fig. 4:**
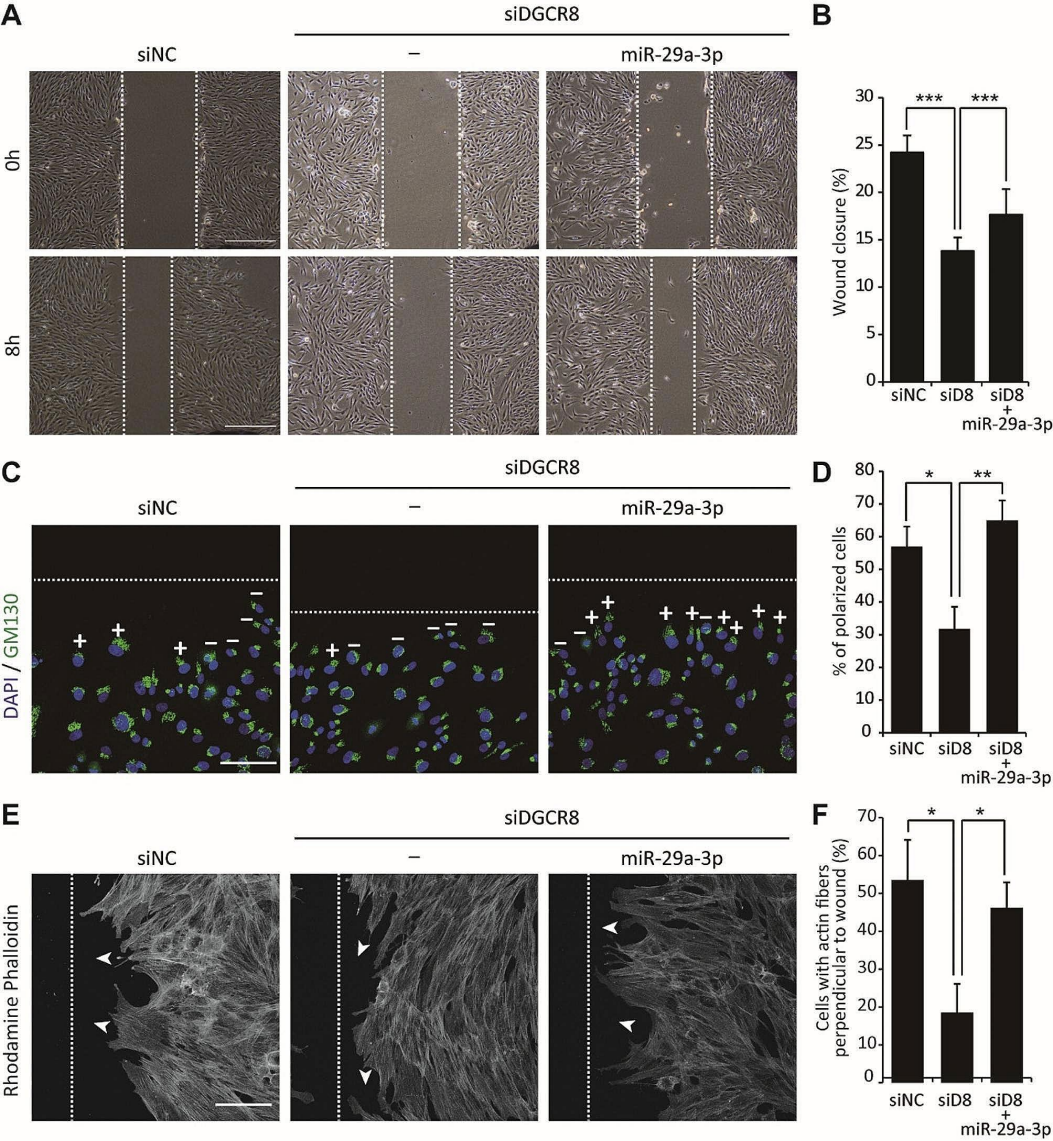
miR-29a-3p modulates hMSC polarization during wound healing. (**A**) Representative images of a wound healing assay of siNC, siDGCR8, and siDGCR8 overexpressing miR-29a-3p (scale bars, 500 μm). (**B**) The percentage of wound closure was calculated as follows: (*A*_*0*_ − *A*_*8*_)/*A*_*0*_. (*A*_*0*_ is the initial (0 h) wound area, and *A*_*8*_ is the wound area after 8 h). (siNC; *n* = 26 wells, siDGCR8; *n* = 26 wells, siDGCR8 + miR-29a-3p; *n* = 19 wells, ***P < 0.001 by Student’s two-tailed t-test). (**C**) Representative immunofluorescence images of Golgi (GM130, green) and nucleus (DAPI, blue) in hMSCs 4 h after wounding. (+) polarized; (–) non-polarized. Scale bars, 100 μm. (**D**) Percentage of polarized hMSCs at the wound edge (siNC; *n* = 53 cells, siDGCR8; *n* = 87 cells, siDGCR8 + miR-29a-3p; *n* = 84 cells, *P < 0.05 and **P < 0.01 by Student’s two-tailed t-test). (**E**) Representative immunofluorescence images of F-actin in hMSCs 4 h after wounding. White arrowheads indicate the orientation of actin fibers. Scale bars, 100 μm. (**F**) Percentage of hMSCs with actin fibers perpendicular to the wound (siNC; *n* = 49 cells, siDGCR8; *n* = 76 cells, siDGCR8 + miR-29a-3p; *n* = 98 cells, *P < 0.05 by Student’s two-tailed t-test). “siD8” indicates siRNA targeting DGCR8.

To determine how miR-29a-3p influences the stepwise process of hMSC migration at the cellular level, we first examined its role in cell polarization and cytoskeletal dynamics. In a wound healing assay, DGCR8 gene knockdown led to reduced wound closure. The decrease in migration was significantly restored upon miR-29-3p overexpression (Fig. 4A, B). The initial establishment of polarization and repositioning of the Golgi apparatus towards the leading edge are essential for cell migration during wound healing [28, 29]. Polarization of the Golgi apparatus was examined by GM130 staining, within a 120° sector facing the wound. Unlike control cells, most DGCR8-knockdown cells in the foremost row were unpolarized (Fig. 4C, depicted as “–”). However, the reintroduction of miR-29a-3p reorganized the Golgi apparatus. Specifically, 56.94% of siNC control cells exhibited a polarized Golgi, compared to only 31.77% of DGCR8-knockdown cells, while 64.98% of miR-29a-3p–overexpressing cells displayed polarization (Fig. 4D). These results demonstrated the crucial role of miR-29a-3p in restoring Golgi polarization in DGCR8-knockdwon cells. Cell polarization in response to wound healing also involves a reorganization of the actin cytoskeleton at the leading edge [30]. At 4 h post-wounding, actin fibers were organized perpendicular to the wound edge in both control and miR-29a-3p–overexpressing hMSCs (Fig. 4E, arrowhead indicated). By contrast, actin fibers in DGCR8-knockdown cells were predominantly oriented parallel to the wound edge. The percentage of cells at the wound edge with a polarized distribution of F-actin was 53.54% in control cells, 18.51% in DGCR8-knockdown cells, and 46.18% in miR-29a-3p overexpressing cells (Fig. 4F). These results showed that miR-29a-3p restores not only Golgi polarization but also the proper orientation of actin fibers, both of which are required for effective cell migration during wound healing.

### miR-29a-3p modulates actomyosin contractility and cellular traction forces in hMSCs

**Fig. 5:**
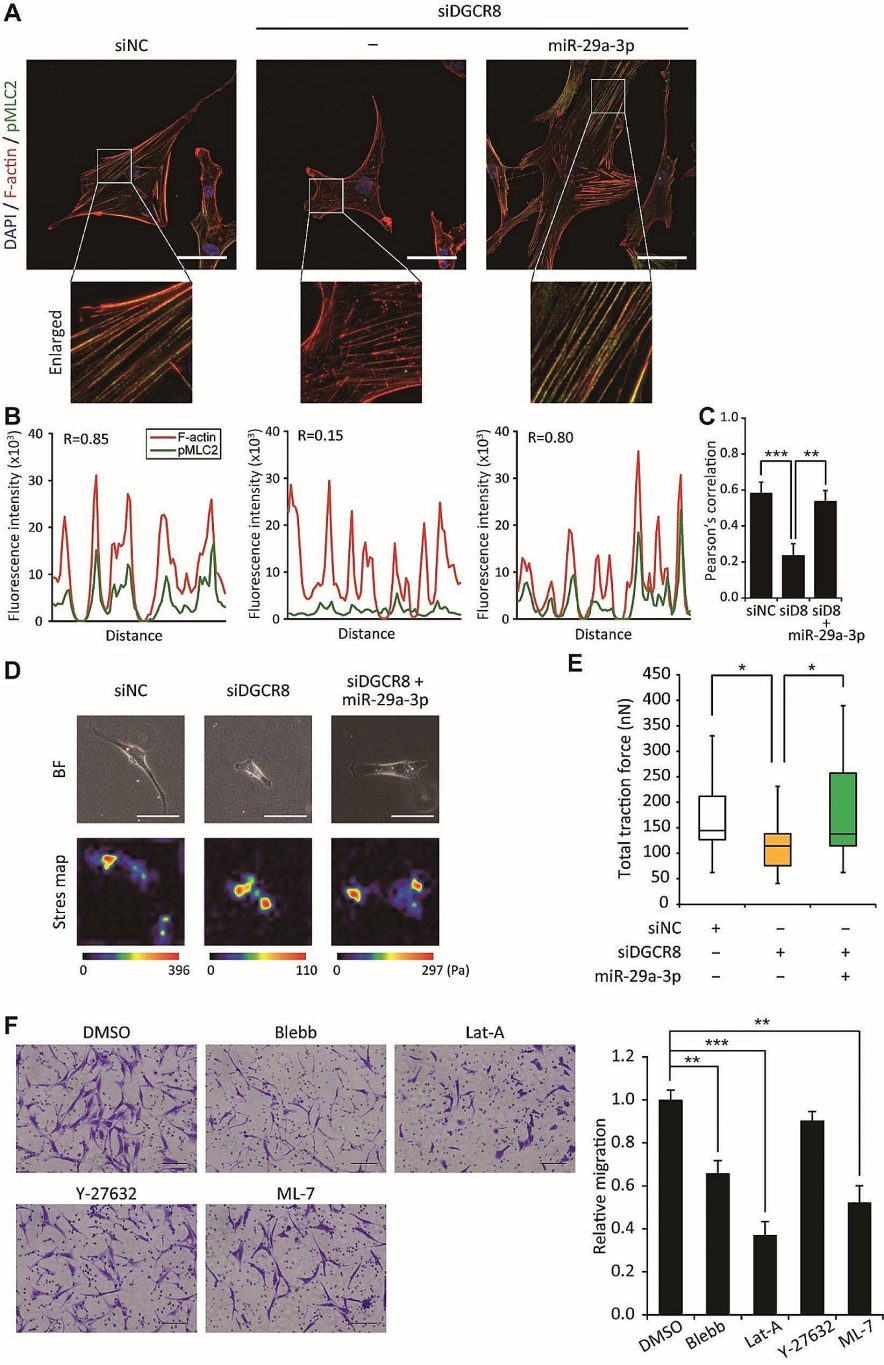
miR-29a-3p regulates actomyosin contractility and cellular traction force in hMSCs. (**A**) Representative fluorescence images of F-actin (red) and pMLC2 (green). A higher-magnification image shows actin and pMLC2. Scale bars, 50 μm. (**B**) Intensity profiles of F-actin (red) and pMLC2 (green) in siNC or siDGCR8 hMSCs with or without miR-29a-3p overexpression. (**C**) Pearson’s correlation coefficients of F-actin and pMLC2 (siNC; *n* = 19 cells, siDGCR8; *n* = 22 cells, siDGCR8 + miR-29a-3p; *n* = 27 cells, **P < 0.01 and ***P < 0.001 by Student’s two-tailed t-test). “siD8” indicates siRNA targeting DGCR8. (**D**) Representative images and stress maps by traction force microscopy of DGCR8-knockdown cells with or without miR-29a-3p forced expression. Scale bars, 50 μm. Force scale bar is in Pascals (Pa). (**E**) Total traction force of control and DGCR8-knockdown cells with or without miR-29a-3p overexpression. Central box, first to third quartile; middle line, median. (siNC; *n* = 20 cells, siDGCR8; *n* = 17 cells, siDGCR8 + miR-29a-3p; *n* = 17 cells, *P < 0.05 by Student’s two-tailed t-test). (**F)** Representative images show a Transwell migration assay in siDGCR8 hMSCs overexpressing miR-29a-3p and treated with the non-muscle myosin II inhibitor blebbistatin (blebb) (10 µM), the actin polymerization inhibitor latrunculin A (Lat-A) (0.1 µM), the Rho-associated protein kinase (ROCK) inhibitor Y-27632 (10 µM), or the myosin light chain kinase inhibitor ML-7 (10 µM). Scale bars represent 100 μm. Migrated cells were quantified and statistically analyzed (error bars indicate standard errors of the mean from four experiments, **P < 0.01, ***P < 0.001 by Student’s two-tailed t-test)

Having established the role of miR-29a-3p in cell polarization, we next investigated the impact of miR-29a-3p on actomyosin contractility and cellular traction, which are required for cell migration and reflect a balance between adhesion and contraction [28, 31, 32]. To address the impact of miR-29a-3p on contractility during cell migration, we monitored active pMLC (Thr18/Ser19). The level of pMLC2 was decreased in DGCR8-knockdown cells but was restored upon miR-29a-3p overexpression (Fig. 5A). Notably, F-actin and pMLC2 staining showed a significant non-overlapping pattern in DGCR8-knockdown cells (Fig. 5B). These abnormal distributions were reversed by miR-29a-3p, as indicated by the co-immunolocalization of pMLC2 with actin fibers and Pearson’s correlation coefficient (Fig. 5C). Because actomyosin-mediated contractility generates the mechanical stress necessary for cell migration [33] and pMLC2 immunofluorescence does not represent the magnitude and distribution of cellular traction forces, actomyosin contractility was quantified by measuring traction forces via TFM (Fig. 5D). Compared to control siNC-transfected cells, DGCR8-knockdown hMSCs exhibited a lower total traction force, consistent with the pMLC2 results (*P* < 0.05, Fig. 5D, E). Overexpression of miR-29a-3p in siDGCR8-transfected cells increased the contractile force to normal levels (*P* < 0.05, Fig. 5D, E). Therefore, miR-29a-3p influences actomyosin contractility and the generation of traction forces in hMSCs.

The mechanisms underlying the activity of miR-29a-3p during cellular migration were investigated using the pharmacological inhibitors blebbistatin (blebb, a non-muscle myosin II inhibitor), latrunculin A (Lat-A, an actin polymerization inhibitor), Y-27632 (a Rho-associated, coiled-coil containing protein kinase (ROCK) inhibitor), and ML-7 (a myosin light chain kinase inhibitor). Despite miR-29a-3p overexpression in a DGCR8 knockdown background, cell migration was significantly inhibited by blebb, Lat-A, and ML-7, but not by Y-27632 (Fig. 5F). This result suggests that the promotion of cellular migration by miR-29a-3p is dependent on the actin cytoskeleton and pMLC2.

### miR-29a-3p modulates focal adhesion and actin cytoskeleton in hMSCs

**Fig. 6:**
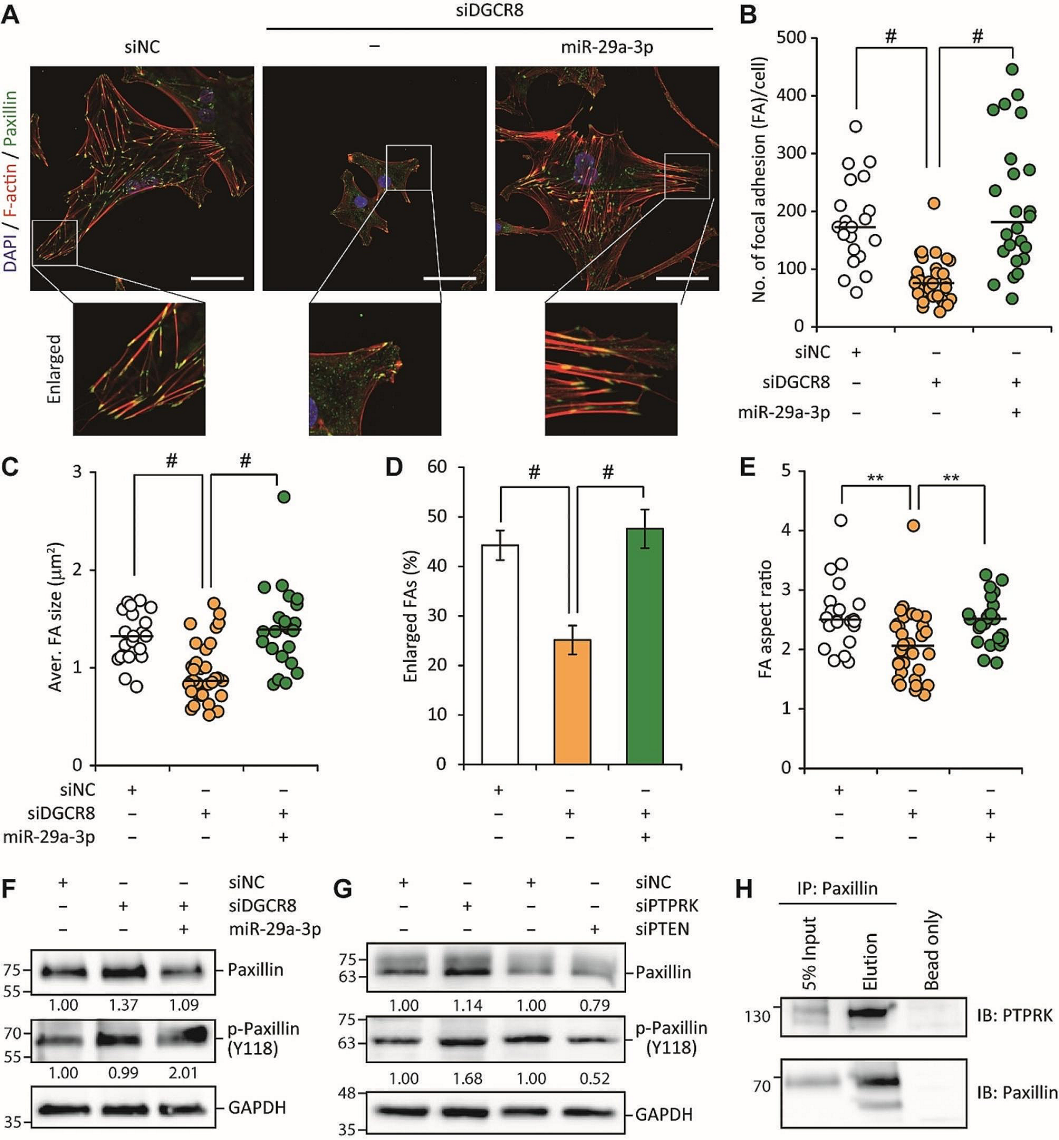
miR-29a-3p modulates focal adhesion formation and maturation by regulating PTPRK and paxillin in hMSCs. (**A**) Representative immunofluorescence images of actin cytoskeleton (F-actin, red) and focal adhesions (paxillin, green) in siNC or siDGCR8 hMSCs with or without miR-29a-3p overexpression. A higher-magnification image shows actin and paxillin. Scale bars, 50 μm. (**B**) Number of focal adhesions per cell, (**C**) average size of focal adhesions per cell, (**D**) percentage of cells with enlarged focal adhesions (greater than the median focal adhesion size), and (**E**) aspect ratio of focal adhesions (siNC; *n* = 21 cells, siDGCR8; *n* = 35 cells, siDGCR8 + miR-29a-3p; *n* = 24 cells; lines, medians, **P < 0.01 and ^#^*P* < 0.0001 by Student’s two-tailed t-test). (**F**) Western blotting of the levels of paxillin and pY118-paxillin (p-Paxillin) in siNC or siDGCR8 hMSCs with or without miR-29a-3p overexpression. GAPDH is the loading control. (**G**) Western blotting of the levels of paxillin and p-Paxillin in siNC, siPTPRK, and siPTEN hMSCs. (**H**) Co-immunoprecipitation of paxillin and PTPRK in immortalized hMSCs. Immunoprecipitation was performed using an anti-paxillin antibody, and precipitated proteins were examined by western blotting using anti-PTPRK and anti-paxillin antibodies

Building on our findings regarding actomyosin contractility, we focused on the role of miR-29a-3p in regulating FAs and the actin cytoskeleton, both of which are also critical for cellular migration [32]. Compared to siNC-transfected hMSCs, the expression of rhodamine phalloidin, an actin cytoskeleton marker, and of paxillin, a FA marker, were reduced in siDGCR8-transfected cells. In siNC-transfected control cells, paxillin showed typical elongated staining aligned with F-actin at the peripheral region (Fig. 6A). However, in DGCR8-knockdown hMSCs, paxillin was smaller, more dispersed, and randomly distributed throughout the cell. Overexpression of miR-29a-3p in siDGCR8-transfected cells fully restored the intensity and distribution of paxillin and F-actin (Fig. 6A, see enlarged images). The absolute number of FAs per cell and their size were significantly reduced in DGCR8-knockdown hMSCs compared to the control. This reduction was reversed by miR-29a-3p overexpression (Fig. 6B, C). FA morphology was then characterized by quantifying the proportion of enlarged FAs per cell and their aspect ratios (the ratio of the major and minor axes). Consistent with the abnormal FAs in DGCR8-knockdown cells, the percentages of enlarged FAs and their aspect ratios were significantly decreased. These aberrations were reversed by miR-29a-3p (Fig. 6D, E). Paxillin is an FA-associated adaptor protein whose function and localization are regulated by phosphorylation at multiple Tyr and Ser residues [34]. We therefore examined the phosphorylation of paxillin (pY118-paxillin), an essential modification for cell migration, using a phospho-specific pY118-paxillin antibody. miR-29a-3p-overexpression siDGCR8 hMSCs exhibited a significant increase in pY118-paxillin (Fig. 6F). Because miR-29a-3p directly represses the expression of PTPRK and PTEN phosphatases, we explored the effect of PTPRK and PTEN knockdown on paxillin and pY118-paxillin levels. Interestingly, PTPRK knockdown in hMSCs increased the level of pY118-paxillin, whereas PTEN knockdown by siRNA reduced the levels of paxillin and pY118-paxillin (Fig. 6G). Next, we examined the interactions between endogenous paxillin and PTPRK by co-immunoprecipitation; paxillin co-precipitated with PTPRK (Fig. 6H). These results indicate that miR-29a-3p regulates FAs during cell migration, at least in part by modulating PTPRK and paxillin.

### miR-29a-3p enhances hMSC migration in vivo

**Fig. 7:**
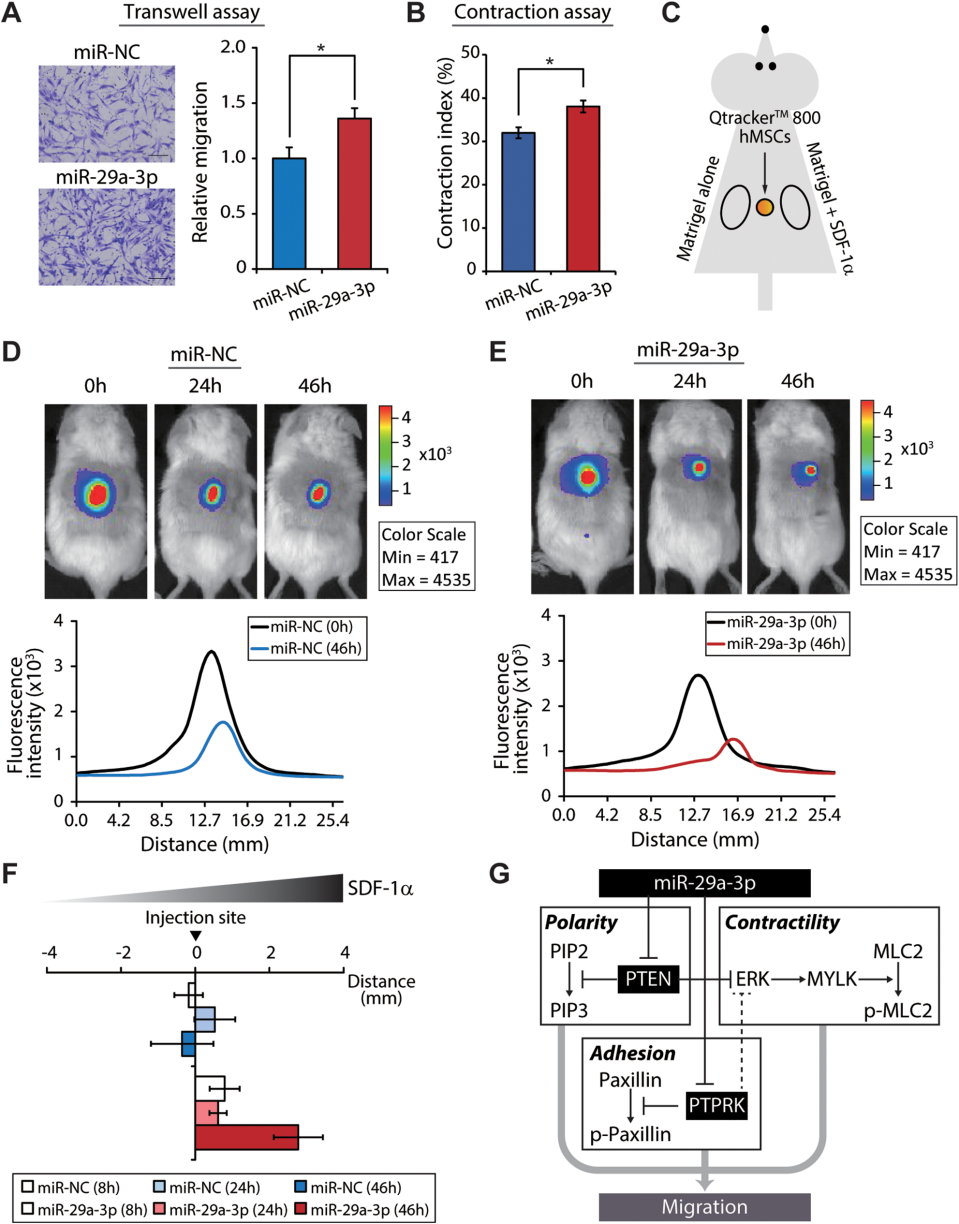
miR-29a-3p enhances hMSC migration in vivo. (**A**) Representative images of a Transwell migration assay of hMSCs with control or miR-29a-3p overexpression. SDF-1α was used as the chemoattractant. Scale bars, 100 μm. Migrated cells were enumerated and statistically analyzed (error bars indicate standard errors of the mean of four experiments, *P < 0.05 by Student’s two-tailed t-test). (**B**) Gel contraction by miR-NC and miR-29a-3p overexpression hMSCs (error bars indicate standard errors of the mean of three experiments, *P < 0.05 by Student’s two-tailed t-test). (**C**) Schematic of subcutaneously implanted Matrigel plugs and injected cells in immunocompromised mice. (**D**,** E**) (Top) Qtracker™ 800-labeled hMSCs with or without miR-29a-3p overexpression were traced in vivo using the IVIS Spectrum In Vivo Imaging System at 0, 24, and 46 h. (Bottom) Fluorescence intensity profiles, along with injection sites. (**F**) Labeled cell distribution at the indicated time points (error bars indicate standard errors of the mean of two or three experiments, mice, *n* = 3/group, miR-29a-3p group at 46 h, *n* = 2). (**G**) A working model of the orchestration of cellular migration by miR-29a-3p, encompassing modulation of the polarization, adhesion, and contractility of hMSCs.

Given that miR-29a-3p influences nearly every key stage of cellular migration in vitro, its function in vivo was investigated after a Transwell migration assay confirmed that its overexpression in wild-type immortalized hMSCs (Fig. S5A) enhances cell migration towards SDF-1α, a chemokine released during inflammation or injury [23, 35, 36] (Fig. 7A). This finding was further supported by a collagen gel contraction assay, which provides insights into interactions between cells and the extracellular matrix (ECM) in 3D environments [37]. Replenishment of miR-29a-3p in hMSCs significantly increased their ability to contract collagen-rich gels (Fig. 7B), consistent with the observed increase in pMLC2 in hMSCs due to miR-29a-3p overexpression in vitro (Fig. 5). The enhanced migration of hMSCs caused by miR-29a-3p overexpression in vivo was demonstrated in an SDF-1α-releasing Matrigel plug assay in immunocompromised mice, creating a chemoattractant gradient to attract hMSC. Matrigel plugs, with or without SDF-1α, were subcutaneously implanted into the right and left sides of NOD/SCID mice, respectively [22]. Qtracker™ 800-labeled miR-29a-3p overexpressing cells and miR-NC-transfected cells were subcutaneously injected at the center and tracked using the IVIS Spectrum Imaging System (Fig. 7C). Before injection, the similar viabilities of control mimic- and miR-29a-3p–transfected hMSCs was confirmed (Fig. S5B). The mice were imaged by IVIS at 0 h to identify labeled hMSCs and at 24 and 46 h to detect MSC migration towards the Matrigel plugs. Compared to control miR-NC-transfected cells, which exhibited marginal migration towards the Matrigel plug with SDF-1α (Fig. 7D, top), miR-29a-3p-overexpression hMSCs showed significantly increased migration (Fig. 7E, top). Fluorescence intensity profiles indicated that miR-29a-3p enhanced the in vivo migration of hMSCs (Fig. 7D, E, bottom). Cell migration was enhanced to the greatest degree in miR-29a-3p-overexpression hMSCs at 46 h post-injection (Fig. 7F). Therefore, miR-29a-3p enhances cell migration in vitro and in vivo.

## Discussion

Based on a comprehensive approach involving global miRNA knockdown by siDGCR8, bioinformatics analysis, and experimental validation, our study provides strong evidence of the critical role of miR-29a-3p in hMSC migration both in vitro and in vivo. Initial investigations revealed pathways enriched in DGCR8-knockdown hMSCs related to cellular assembly and organization, such as Golgi apparatus orientation, FA formation, and actin cytoskeleton organization. These findings correlated with the morphological changes and impaired migration observed in DGCR8-depleted hMSCs. By contrast, miR-29a-3p overexpression markedly enhanced cellular migration, aligning with previous reports and suggesting that studies combining DGCR8 knockout with miRNA rescue can provide profound insights into miRNA functionality [38], including the identification of multiple targets and the overlapping roles of miRNAs in migration and other essential cellular processes.

Previous studies have implicated miR-29a-3p in various cancers, where it acts primarily as a repressor but also activates migration and invasion depending on the circumstances [27]. In line with its activator role, our study newly showed that miR-29a-3p enhances hMSC migration. These results suggest that the functions of miRNAs can vary significantly depending on the cell type and specific biological conditions.

Cellular migration involves a series of interconnected and repetitive processes important for embryonic development, angiogenesis, nerve growth, inflammation, stem cell homing, and metastasis [39–42]. Our findings indicate that miR-29a-3p affects cell motility by influencing various stages of migration. The enrichment map analysis based on DGCR8-knockdown and control cells revealed significant enrichment of a subnetwork of phosphatases (Fig. S6), emphasizing the importance of the miR-29a-3p-mediated suppression of PTPRK and PTEN, which in turn affects multiple downstream signaling components (Fig. 7G). By regulating PTEN, miR-29a-3p influences chemotaxis and the actin cytoskeleton, which are critical for migration. miR-29a-3p also stabilizes Golgi apparatus polarization in addition to modulating actomyosin contractility and cellular traction forces, required for effective cell movement.

Unlike PTEN, the function of PTPRK during cell migration was unclear. Our findings show that miR-29a-3p directly represses PTPRK, leading to the accumulation of p-paxillin. This regulatory mechanism depends on the direct interaction between PTPRK and paxillin (Fig. 7G). Because PTPRK negatively regulates ERK, the repression of PTPRK by miR-29a-3p could affect actomyosin contractility [43–45]. Again, this underscores the context-dependent nature of miRNA action, with miRNAs playing different roles based on the cellular environment and target interactions.

Considering the roles of FAK and pFAK in FA dynamics and cell motility, we monitored their levels in siNC, siDGCR8, and siDGCR8 cells with miR-29a-3p overexpression using Western blotting. Interestingly, miR-29a-3p expression reduced the pFAK/FAK ratio (data not shown). Because the phosphorylation of FAK is typically associated with the rapid turnover of FAs and enhanced cell migration, this finding is inconsistent with previous reports. We speculate that cellular migration in hMSCs in our experimental setting does not rely on FAK dynamics.

While our study provides significant insights into the role of miR-29a-3p in hMSC migration, its limitations should also be noted. First, the in vivo experiments were conducted in immunocompromised mice, which might not fully replicate the complex immune environment in human tissues. Second, while PTPRK and PTEN were identified as key targets of miR-29a-3p, there may be other as-yet-unidentified targets that contribute to the observed effects. Third, compared to a 2D surface, 3D cellular migration involves complex cellular responses to multiple environmental pressures. Our collagen contraction assay suggested that miR-29a-3p has a direct and/or indirect function in remodeling the ECM, which is critical for cellular permeation of extracellular barriers but this was not further investigated. Future studies should address these limitations by exploring miR-29a-3p function in more complex and clinically relevant models, as well as identifying other potential miR-29a-3p targets.

## Conclusions

Our findings suggest that miR-29a-3p orchestrates the migration of hMSCs by directly regulating two key phosphatases PTPRK and PTEN. These regulatory cascades, involving miRNA modulation of critical phosphatases and their downstream targets, not only amplify signaling pathways but also enable the fine-tuning of cellular responses. Consequently, this regulatory network significantly promotes the migration of hMSCs in vitro and in vivo. These insights into the miRNA-mediated regulation of hMSC migration highlight the therapeutic potential of targeting specific miRNAs. Modulating miR-29a-3p could enhance tissue repair and regeneration, offering promising new strategies for clinical interventions aimed at restoring cell function and improving outcomes in regenerative medicine.

### Electronic supplementary material

Below is the link to the electronic supplementary material.

Supplementary Material 1

## Data Availability

No datasets were generated or analysed during the current study.
