# Supplementary material for: miR-29a-3p orchestrates key signaling pathways for enhanced migration of human mesenchymal stem cells
Source: Cell Commun Signal. 2024 Jul 17;22:365. doi: 10.1186/s12964-024-01737-0 (PMC11256664; doi:10.1186/s12964-024-01737-0)
Supplement: Supplementary file 1 — Supplementary Material 1 [file 12964_2024_1737_MOESM1_ESM.docx]

**Supplementary Information**

**Table S1. Primers used for construction of PTPRK luciferase reporters.**

| **Name** | **Sequence** |
| --- | --- |
| PTPRK WT-F | 5'-GTGTCTCGAGTTGGGTGAGACTCTT-3' |
| PTPRK WT-R | 5'-ATGCGGCCGCTAACTGTTATAATTA-3' |
| PTPRK M-F | 5'-GATTGCCAGCTCATGACATGATACTTATAAAGATTTAATTAAAG-3' |
| PTPRK M-R | 5'-CTTTAATTAAATCTTTATAAGTATCATGTCATGAGCTGGCAATC-3' |

**Table S2. Primers used for quantitative RT-PCR analysis.**

| **Gene** | **Forward** | **Reverse** |
| --- | --- | --- |
| GAPDH | GAAGGTGAAGGTCGGAGTCA | GACAAGCTTCCCGTTCTCAG |
| PTPRK | GACGATTATCCACTGCCTAAATG | ACCATTTCAACAACGATGCCT |
| PTEN | CGACGGGAAGACAAGTTCAT | AGGTTTCCTCTGGTCCTGGT |
| DGCR8 | GTCAAGCAGGAGACATCGGA | GAGTCTCCTCCCTTTCCTCA |

**Table S3. Principle molecular and cellular functions associated with DGCR8 knockdown.**

| **Molecular and cellular functions** | ***p-*value range** | **No. of molecules** |
| --- | --- | --- |
| **Cell death and survival** | **6.39E-04–1.70E-13** | **610** |
| Necrosis | 1.70E-13 | 477 |
| Apoptosis | 5.25E-11 | 450 |
| Cell viability of tumor cell lines | 6.30E-10 | 199 |
| **Gene expression** | **6.09E-04–4.81E-12** | **374** |
| Expression of RNA | 4.81E-12 | 362 |
| Transcription | 1.77E-11 | 341 |
| Transcription of RNA | 6.08E-10 | 284 |
| **Cellular assembly and organization** | **6.99E-04–8.15E-11** | **444** |
| Organization of cytoplasm | 8.15E-11 | 298 |
| Organization of cytoskeleton | 2.41E-08 | 264 |
| Microtubule dynamics | 4.93E-08 | 235 |
| **Cellular function and maintenance** | **6.16E-04–8.15E-11** | **506** |
| Organization of cytoplasm | 8.15E-11 | 298 |
| Organization of cytoskeleton | 2.41E-08 | 264 |
| Microtubule dynamics | 4.93E-08 | 235 |
| **Cell cycle** | **6.36E-04–1.06E-08** | **295** |
| Interphase | 1.06E-08 | 140 |
| Senescence of cells | 8.94E-07 | 72 |
| Cell cycle progression | 1.12E-06 | 179 |

**Table S4. A list of the 62 potential target genes of miR-29a-3p.**

| **PROBE_ID** | **SYMBOL** | **SEARCH_KEY** | **siGFP** | **siDGCR8** | **Fold** |
| --- | --- | --- | --- | --- | --- |
| ILMN_1714108 | TP53INP1 | NM_033285.2 | 198.32 | 365.65 | 1.840 |
| ILMN_1775708 | SLC2A3 | NM_006931.1 | 1305.75 | 2310.52 | 1.770 |
| ILMN_1745994 | GAS7 | NM_201432.1 | 130.51 | 220.25 | 1.690 |
| ILMN_1721842 | RYBP | NM_012234.3 | 388.56 | 641.51 | 1.650 |
| ILMN_1802615 | CDK6 | NM_001259.5 | 346.57 | 563.78 | 1.630 |
| ILMN_1714700 | TRIB2 | NM_021643.1 | 145.11 | 233.45 | 1.610 |
| ILMN_1797728 | HMGCS1 | NM_002130.4 | 413.19 | 661.53 | 1.600 |
| ILMN_1801616 | EMP1 | NM_001423.1 | 3029.09 | 4714.86 | 1.560 |
| ILMN_1754279 | FBXW7 | NM_033632.2 | 173.57 | 263.01 | 1.520 |
| ILMN_1764177 | JARID2 | NM_004973.2 | 166.84 | 253.94 | 1.520 |
| ILMN_1801403 | DCUN1D4 | NM_015115.1 | 284.64 | 424.02 | 1.490 |
| ILMN_1770085 | BTG2 | NM_006763.2 | 132.67 | 192.54 | 1.450 |
| ILMN_1800626 | SESN1 | NM_014454.1 | 172.84 | 247.57 | 1.430 |
| ILMN_2320349 | BACE1 | NM_138972.2 | 194.64 | 277.88 | 1.430 |
| ILMN_3245625 | RFX7 | NM_022841.5 | 241.97 | 338.18 | 1.400 |
| ILMN_1652521 | MTMR9 | NM_015458.3 | 207.07 | 279.49 | 1.350 |
| ILMN_1730940 | KLHDC3 | NM_057161.2 | 751.69 | 1014.81 | 1.350 |
| ILMN_1736510 | FOXN2 | NM_002158.2 | 201.5 | 271.03 | 1.350 |
| ILMN_2375002 | MAP4K4 | NM_145687.2 | 321.72 | 432.43 | 1.340 |
| ILMN_1670875 | PPM1D | NM_003620.2 | 148.16 | 194.96 | 1.320 |
| ILMN_1701134 | PTEN | NM_000314.3 | 241.58 | 319.06 | 1.320 |
| ILMN_1760303 | PIK3R1 | NM_181504.2 | 172.8 | 227.49 | 1.320 |
| ILMN_1810962 | PTPRK | NM_002844.2 | 290.22 | 376.89 | 1.300 |
| ILMN_1721921 | BLMH | NM_000386.2 | 142.12 | 182.78 | 1.290 |
| ILMN_1686884 | IL1RAP | NM_002182.2 | 112.83 | 143.97 | 1.280 |
| ILMN_1782609 | STAG2 | NM_006603.3 | 264.78 | 336.86 | 1.270 |
| ILMN_1788213 | FRAT2 | NM_012083.2 | 142.22 | 179.94 | 1.270 |
| ILMN_1674719 | NID1 | NM_002508.1 | 118.52 | 148.54 | 1.250 |
| ILMN_1665335 | DIABLO | NM_138930.2 | 136.27 | 169.01 | 1.240 |
| ILMN_1742427 | POLR1D | NM_152705.1 | 200.58 | 246.88 | 1.230 |
| ILMN_2115696 | USP42 | NM_032172.1 | 207.31 | 256 | 1.230 |
| ILMN_1681016 | SPNS1 | NM_032038.1 | 483.49 | 592.09 | 1.220 |
| ILMN_1743275 | SH3RF3 | XM_938910.1 | 93.55 | 112.78 | 1.210 |
| ILMN_1749253 | TUBD1 | NM_016261.2 | 103.2 | 124.61 | 1.210 |
| ILMN_1778673 | GOLGA7 | NM_001002296.1 | 831.75 | 1008.97 | 1.210 |
| ILMN_3245236 | FBRS | NM_001105079.1 | 356.72 | 430.54 | 1.210 |
| ILMN_1758906 | GNA13 | NM_006572.3 | 354.75 | 426.28 | 1.200 |
| ILMN_1809889 | CCDC117 | NM_173510.1 | 187.92 | 224.72 | 1.200 |
| ILMN_1706376 | OSBP | NM_002556.2 | 482.26 | 573.11 | 1.190 |
| ILMN_1739454 | USP34 | NM_014709.2 | 173.85 | 204.36 | 1.180 |
| ILMN_1800951 | ATXN1 | NM_000332.2 | 212.99 | 249.46 | 1.170 |
| ILMN_1810514 | SLC25A44 | NM_014655.1 | 273.68 | 320.53 | 1.170 |
| ILMN_1804629 | TPK1 | NM_022445.2 | 153.53 | 177.54 | 1.160 |
| ILMN_1657395 | HMGCR | NM_000859.1 | 338.26 | 390.09 | 1.150 |
| ILMN_1696270 | PLAG1 | NM_002655.1 | 106.99 | 123.33 | 1.150 |
| ILMN_1700834 | SLK | NM_014720.2 | 213.68 | 245.74 | 1.150 |
| ILMN_1770892 | YY1 | NM_003403.3 | 1164.64 | 1342.77 | 1.150 |
| ILMN_1781198 | PPP1R3D | NM_006242.3 | 114.59 | 131.81 | 1.150 |
| ILMN_1781691 | TRAK2 | NM_015049.1 | 463.15 | 532.51 | 1.150 |
| ILMN_1808326 | NPAS3 | NM_022123.1 | 96.34 | 110.33 | 1.150 |
| ILMN_1812776 | FBXO28 | NM_015176.1 | 198.55 | 228.6 | 1.150 |
| ILMN_2129273 | STRN | NM_003162.2 | 107.91 | 123.9 | 1.150 |
| ILMN_1677997 | MAZ | NM_002383.1 | 114.22 | 130 | 1.140 |
| ILMN_1772486 | ELF2 | NM_006874.2 | 137.19 | 156.86 | 1.140 |
| ILMN_1792389 | RNF165 | NM_152470.2 | 93.85 | 105.27 | 1.120 |
| ILMN_1726420 | BSND | NM_057176.2 | 84.14 | 93.38 | 1.110 |
| ILMN_1779486 | FAM126B | NM_173822.1 | 114.46 | 126.82 | 1.110 |
| ILMN_1805376 | KCNJ6 | NM_002240.2 | 84.78 | 93.2 | 1.100 |
| ILMN_1689102 | C1QTNF6 | NM_031910.3 | 87.67 | 95.3 | 1.090 |
| ILMN_1736068 | CNOT8 | NM_004779.4 | 124.33 | 135.89 | 1.090 |
| ILMN_1782441 | CDKL2 | NM_003948.2 | 85.73 | 92.43 | 1.080 |
| ILMN_2387303 | SHPRH | NM_173082.2 | 91.69 | 98.91 | 1.080 |

**Table S5. Principle molecular and cellular functions associated with the 62 target genes of miR-29a-3p.**

| **Molecular and cellular functions** | ***p-*value range** | **No. of molecules** |
| --- | --- | --- |
| **Cell death and survival** | **3.93E-02–4.39E-06** | **29** |
| Cell viability | 4.39.E-06 | 19 |
| Cell viability of tumor cell lines | 5.06.E-06 | 17 |
| Cell viability of cervical cancer cell lines | 2.61.E-04 | 6 |
| **Cell morphology** | **2.86E-02–1.23E-04** | **12** |
| Size of fibroblast cell lines | 1.23.E-04 | 2 |
| Orientation of Golgi apparatus | 2.29.E-04 | 2 |
| Loss of neurons in central nervous system | 2.94.E-04 | 2 |
| **Cellular growth and proliferation** | **3.98E-02–1.23E-04** | **22** |
| Size of fibroblast cell lines | 1.23.E-04 | 2 |
| Proliferation of lung cancer cell lines | 5.32.E-04 | 8 |
| Colony formation of adenocarcinoma cell lines | 1.09.E-03 | 2 |
| **Cellular assembly and organization** | **3.86E-02–2.29E-04** | **19** |
| Orientation of Golgi apparatus | 2.29.E-04 | 2 |
| Formation of focal adhesions | 3.96.E-04 | 4 |
| Formation of actin | 6.32.E-04 | 2 |
| **Cellular development** | **3.98E-02–2.46E-04** | **23** |
| Differentiation of adipose cell lines | 2.46.E-04 | 3 |
| Differentiation of adipocytes | 4.63.E-04 | 4 |
| Proliferation of lung cancer cell lines | 5.32.E-04 | 8 |

**
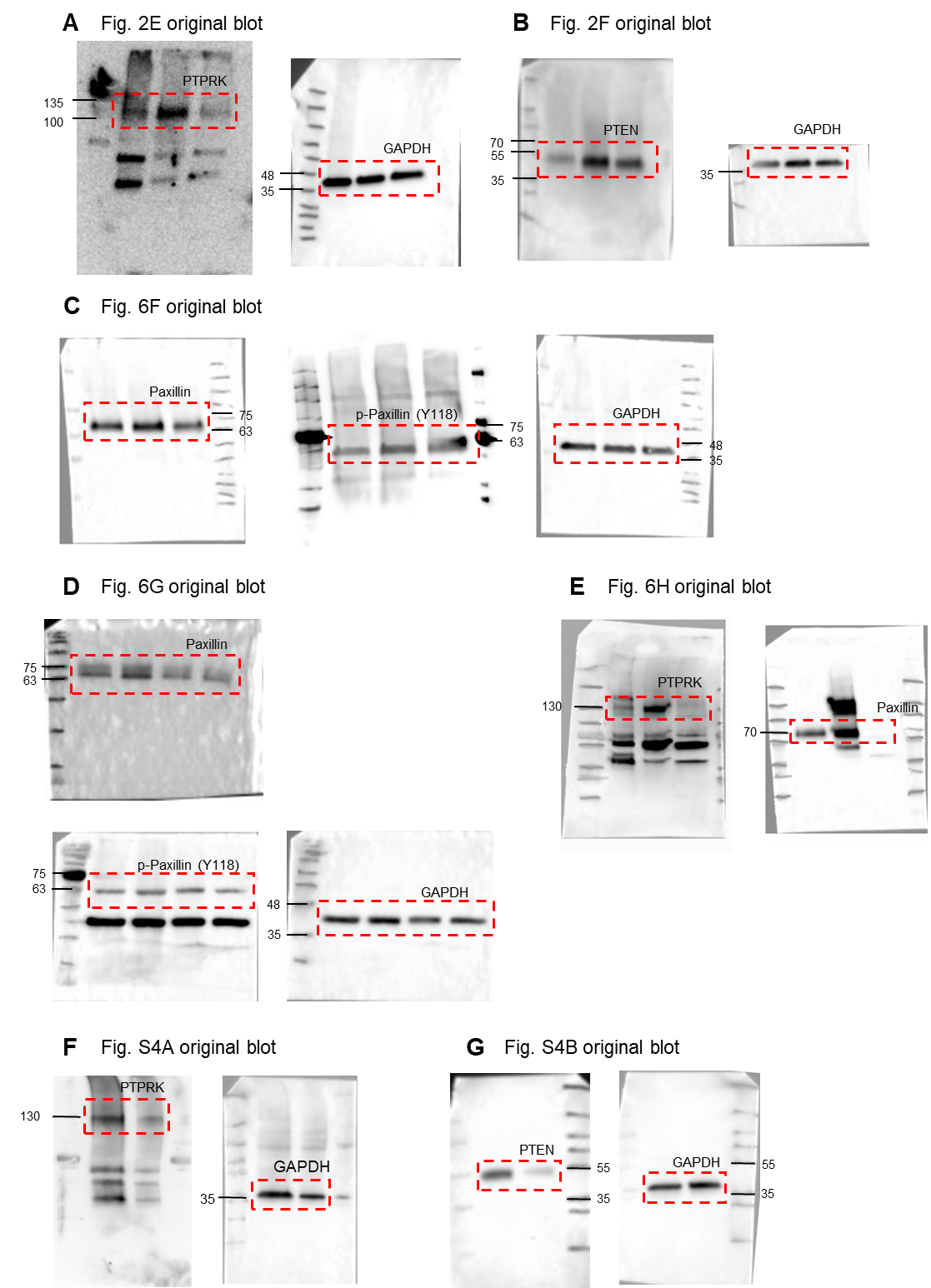
**

**Fig. S1. Images of the Western blots used for quantification of protein levels.** Red dotted squares indicate lanes shown in the main and supplementary figures.


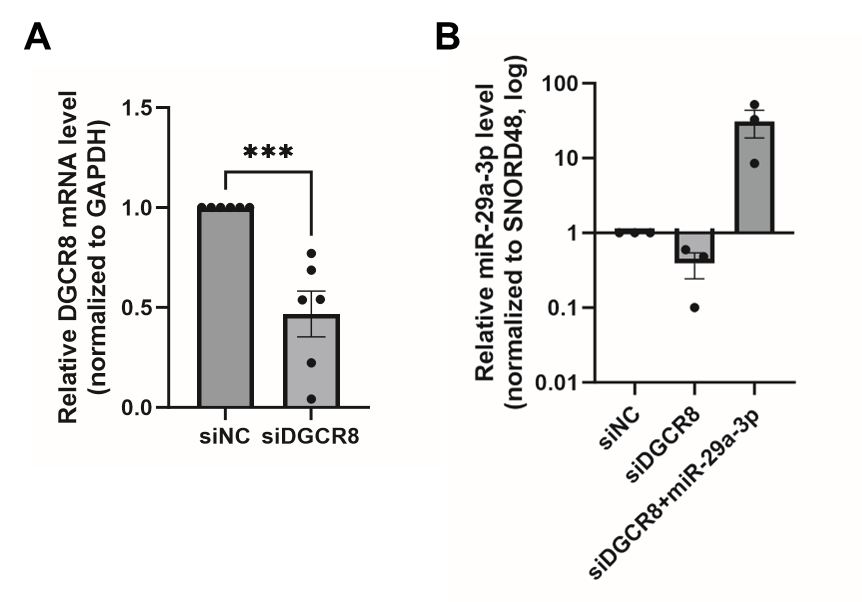


**Fig. S2. Relative expression levels of DGCR8 mRNA and miR-29a-3p.** (**A**) DGCR8 mRNA level quantified via qRT-PCR and normalized to the GAPDH mRNA level (error bars indicate standard errors of the mean of six experiments. ****P* < 0.001 in Student’s two-tailed t-test). (**B**) miR-29a-3p level as determined by qRT-PCR and normalized to the SNORD48 level (error bars indicate standard errors of the mean of three experiments).


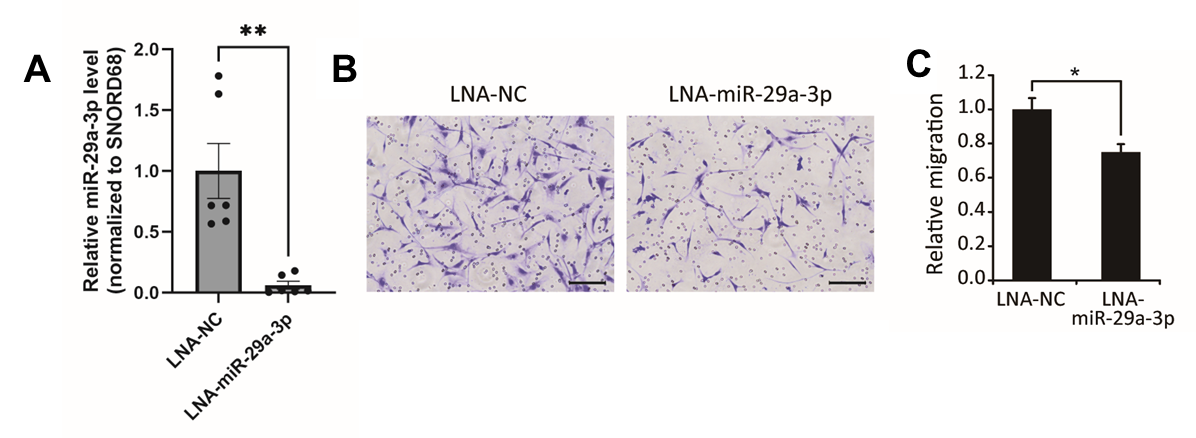


**Fig. S3. Inhibition of miR-29a-3p expression in hMSCs** (**A**) miR-29a-3p level as determined by qRT-PCR and normalized to the SNORD68 level (error bars indicate standard errors of the mean of six experiments, ***P* < 0.01 by Student’s two-tailed t-test). (**B**) Representative images of a Transwell migration assay of negative control (LNA-NC) and LNA-miR-29a-3p transfected hMSCs. Scale bars, 100 μm. (**C**) Migrated cells were enumerated and statistically analyzed (error bars indicate standard errors of the mean of four experiments, **P* < 0.05 by Student’s two-tailed *t*-test).


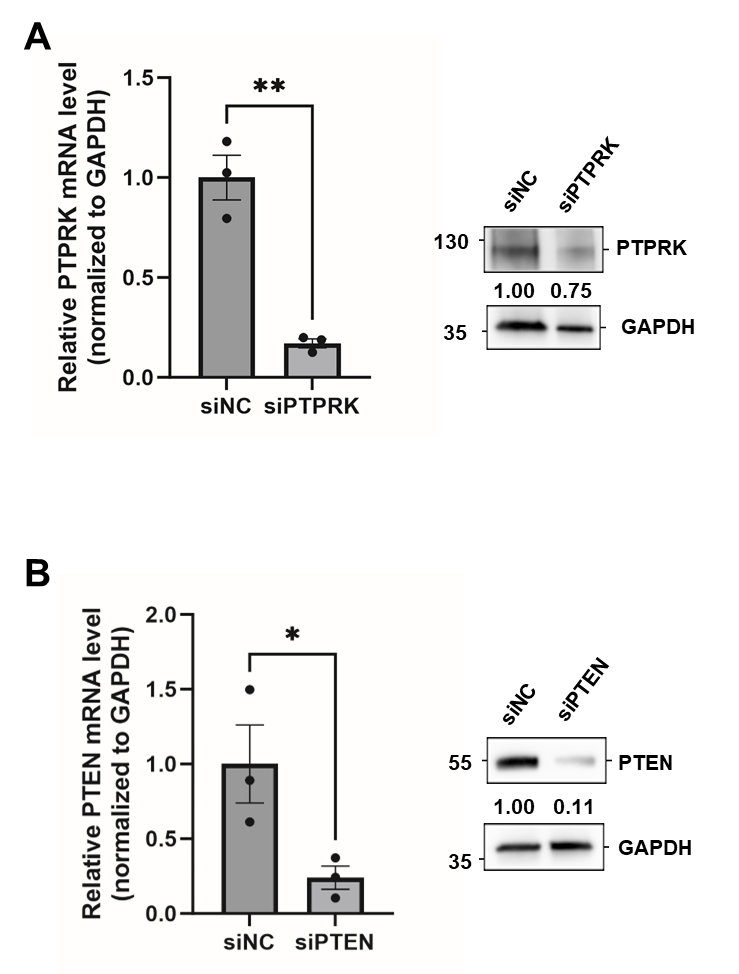


**Fig. S4. Quantification of PTPRK and PTEN levels.** mRNA and protein levels of (**A**) PTPRK and (**B**) PTEN as determined by qRT-PCR and Western blotting, respectively. Data were normalized to the GAPDH mRNA or protein level (error bars indicate standard errors of the mean of three experiments, **P*<0.05 and ***P* < 0.01 by Student’s two-tailed t-test).


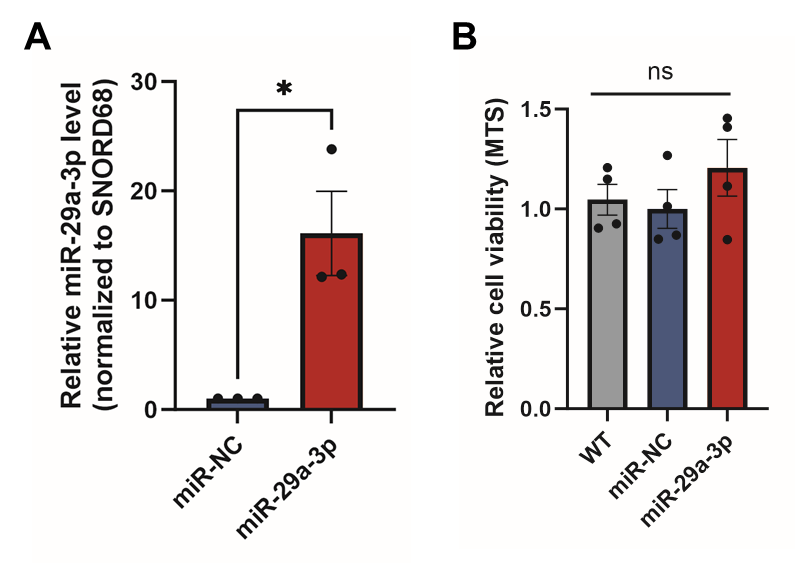


**Fig. S5.** **Overexpression of miR-29a-3p in hTERT-hMSCs.** (**A**) miR-29a-3p level as determined by qRT-PCR and normalized to the SNORD68 level (error bars indicate standard errors of the mean of three experiments, **P* < 0.05 by Student’s two-tailed t-test). (**B**) Cell viability as measured by MTS assay and normalized to the wild-type (WT) (error bars indicate standard errors of the mean of four experiments; ns, not significant by Student’s two-tailed t-test).


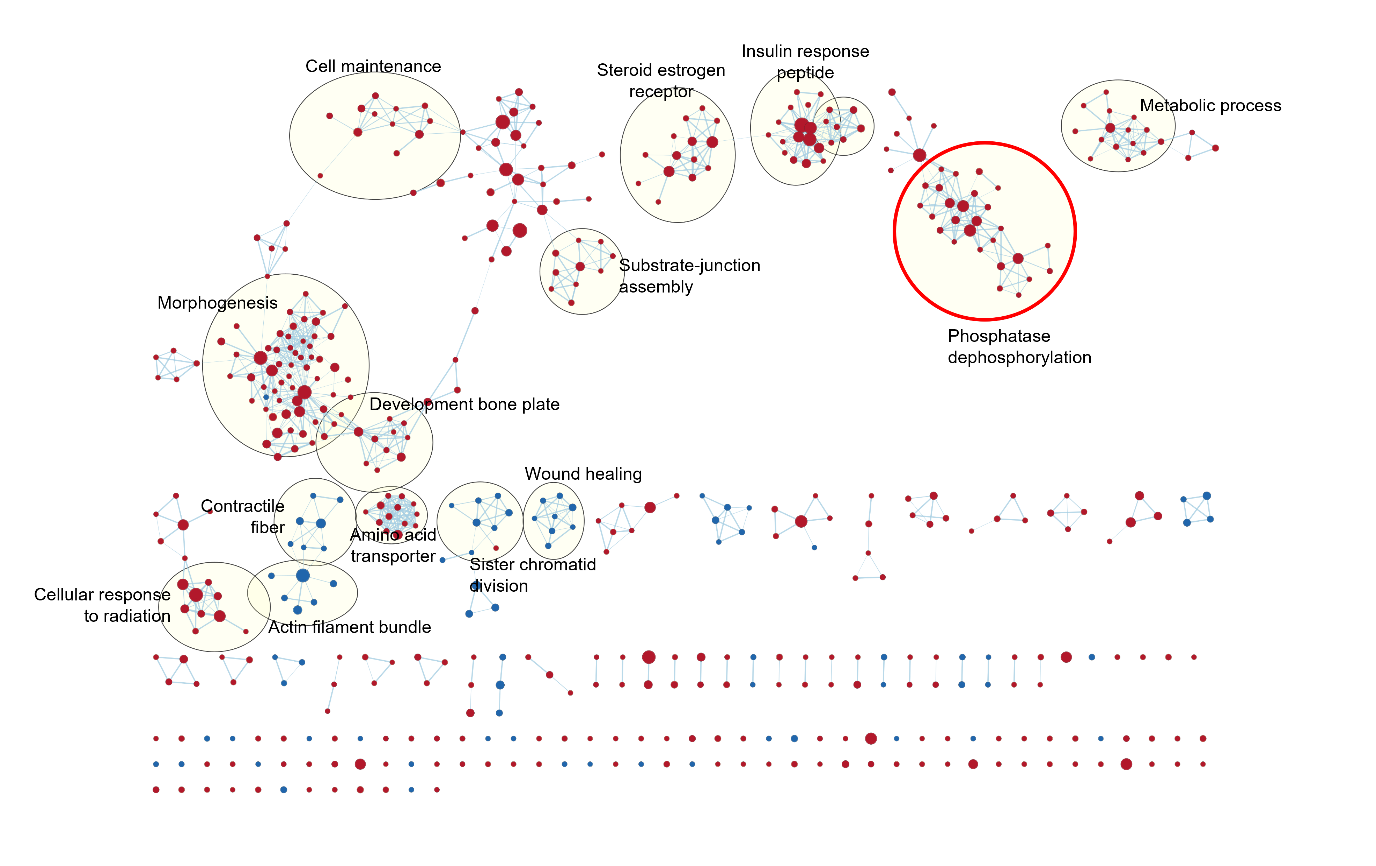


**Fig. S6. GSEA Enrichment map.** Gene ontologies of siDGCR8- vs. siGFP-transfected hMSCs (FDR < 0.25, overlap coefficient 0.5) using GSEA were visualised using EnrichmentMap and AutoAnnotate application in Cytoscape 3.10.1. Each node represents a single gene set, node size corresponds to the number of genes, and node colour the enrichment score (red; upregulated in siDGCR8, blue; downregulated in siDGCR8). The overlap between pathways is represented by blue lines.
